# The Yeast HMGB Protein Hmo1 Is a Multifaceted Regulator of DNA Damage Tolerance

**DOI:** 10.3390/ijms26073255

**Published:** 2025-04-01

**Authors:** Jinlong Huo, Anhui Wei, Na Guo, Ruotong Wang, Xin Bi

**Affiliations:** 1 Department of Biology, University of Rochester, Rochester, NY 14627, USA; jinlonghuo973@163.com (J.H.); weianhui@jlu.edu.cn (A.W.); abas1980@163.com (N.G.); rwang44@u.rochester.edu (R.W.); 2 College of Animal Science and Technology, Yunnan Agricultural University, Kunming 650201, China; 3 Institute of Frontier Medical Sciences, Jilin University, Changchun 130021, China; 4 College of Food Science and Engineering, Jilin University, Changchun 130062, China

**Keywords:** HMGB protein, Hmo1, DNA damage tolerance, DNA replication, DNA damage checkpoint, Htz1, chromatin

## Abstract

The *Saccharomyces cerevisiae* chromosomal architectural protein Hmo1 is categorized as an HMGB protein, as it contains two HMGB motifs that bind DNA in a structure-specific manner. However, Hmo1 has a basic C-terminal domain (CTD) that promotes DNA bending instead of an acidic one found in a canonical HMGB protein. Hmo1 has diverse functions in genome maintenance and gene regulation. It is implicated in DNA damage tolerance (DDT) that enables DNA replication to bypass lesions on the template. Hmo1 is believed to direct DNA lesions to the error-free template switching (TS) pathway of DDT and to aid in the formation of the key TS intermediate sister chromatid junction (SCJ), but the underlying mechanisms have yet to be resolved. In this work, we used genetic and molecular biology approaches to further investigate the role of Hmo1 in DDT. We found extensive functional interactions of Hmo1 with components of the genome integrity network in cellular response to the genotoxin methyl methanesulfonate (MMS), implicating Hmo1 in the execution or regulation of homology-directed DNA repair, replication-coupled chromatin assembly, and the DNA damage checkpoint. Notably, our data pointed to a role for Hmo1 in directing SCJ to the nuclease-mediated *resolution* pathway instead of the helicase/topoisomerase mediated *dissolution* pathway for processing/removal. They also suggested that Hmo1 modulates both the recycling of parental histones and the deposition of newly synthesized histones on nascent DNA at the replication fork to ensure proper chromatin formation. We found evidence that Hmo1 counteracts the function of histone H2A variant H2A.Z (Htz1 in yeast) in DDT possibly due to their opposing effects on DNA resection. We showed that Hmo1 promotes DNA negative supercoiling as a proxy of chromatin structure and MMS-induced DNA damage checkpoint signaling, which is independent of the CTD of Hmo1. Moreover, we obtained evidence indicating that whether the CTD of Hmo1 contributes to its function in DDT is dependent on the host’s genetic background. Taken together, our findings demonstrated that Hmo1 can contribute to, or regulate, multiple processes of DDT via different mechanisms.

## 1. Introduction

Chromatin plays a critical role in the function of the eukaryotic genome by regulating DNA transactions, including replication, recombination, and repair, as well as gene transcription. The basic unit of chromatin is the nucleosome, composed of 147 base pairs of DNA wrapping around a protein core made of two copies each of histones H2A, H2B, H3, and H4 [1]. Nucleosomes are connected by linker DNA sequences to form the primary chromatin structure. The formation of higher order chromatin structure is facilitated by the linker histone H1 that associates with linker DNA [2]. Chromatin compacts and protects DNA but may also hinder DNA transactions. Cells have evolved mechanisms to modify/remodel chromatin to create global or local chromatin environments/states that allow genome functions to take place. One such mechanism is the modulation of chromatin dynamics by the high mobility group (HMG) proteins, an evolutionally conserved group of non-histone chromosomal architectural proteins [3]. HMG proteins include the HMBA, HMGB and HMGN families, of which HMGB family is the largest. An HMGB protein typically consists of one or more HMG-boxes (aka HMGB motifs) and an acidic carboxy-terminal domain (CTD) containing consecutive glutamate and aspartate residues. An HMGB motif is a sequence-nonspecific DNA binding module that recognizes altered DNA structures such as hemicatenane and four-way junctions and can distort DNA via bending and unwinding [3]. The CTD of an HMGB protein regulates its DNA binding properties [3]. In mammals, HMGB proteins are known to interact with nucleosomes to evict the linker histone H1, making chromatin more accessible to other chromatin binding factors [4].

*Saccharomyces cerevisiae* Hmo1 is deemed an HMGB protein, as it contains two HMGB motifs that binds DNA [5,6]. However, unlike canonical HMGB proteins, Hmo1 has a basic, lysine-rich CTD, similar to the linker histone H1. In line with its special domain structure, Hmo1 exhibits functional characteristics of both HMGB proteins and the linker histone H1 [6,7]. On the one hand, like HMGB proteins, Hmo1 destabilizes nucleosomes [8,9] and facilitates the recruitment of gene regulators [10,11]. On the other hand, like linker histones, Hmo1 makes the yeast genome refractory to nuclease digestion possibly by promoting chromatin compaction and rendering it less dynamic [5,12]. Notably, this linker histone-like function of Hmo1 is dependent on its CTD [12]. What determines whether Hmo1 performs HMGB-like or linker histone-like functions in the cell is not clear.

**Figure 1:**
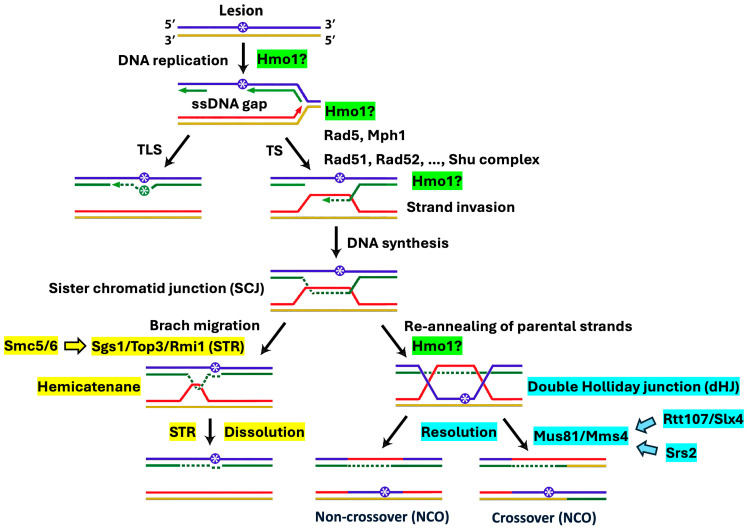
Mechanisms of DNA damage tolerance (DDT). A DNA lesion (asterisk in blue circle) inflicted by genotoxins may stall DNA replication. Uncoupling of DNA polymerase and helicase or re-initiation of DNA synthesis at a distance from the lesion leads to the formation of ssDNA gaps on the leading and lagging strands (a ssDNA gap on lagging strand is shown). Repair/filling of ssDNA gaps can be achieved via the translesion synthesis (TLS) or template switching (TS) pathways that are mechanistically different. SCJ, sister chromatid junction; “Hmo1?” denotes possible position/step of the DDT mechanisms where Hmo1 may act or regulate. See the text for more descriptions.

As an abundant protein that associates with chromatin throughout the cell cycle [13], Hmo1 impacts multiple cellular functions [7]. Hmo1 is important for cell growth, as cells lacking Hmo1 exhibit a severe slow growth phenotype due to a significant lengthening of the doubling time/cell cycle [5]. Hmo1 acts as a transcription factor to promote the expression of genes involved in ribosome biogenesis [7]. Hmo1 was recently found to bind the boundaries of RNA polymerase II transcribed genes and help establish a shared topological configuration of these genes [14]. Hmo1 was also found to affect the repair of double-stranded DNA (dsDNA) breaks (DSBs) by stabilizing chromatin and hindering DNA end resection [15,16]. In addition, there is evidence that Hmo1 regulates cellular tolerance to methyl methanesulfonate (MMS), a genotoxin that methylates DNA and stalls DNA replication [17]. During DNA replication, damage to the template may render leading strand and lagging strand synthesis discontinuous due to the uncoupling of DNA polymerase from DNA helicase or the re-initiation of DNA synthesis at a distance from the lesion [18,19]. This results in single-stranded DNA (ssDNA) gaps in the wake of stalled replication forks, which can be repaired/filled by two mechanistically distinct DNA damage tolerance (DDT) pathways that enable the replisome to bypass DNA lesions and complete DNA replication [20,21] (Figure 1). One mechanism is translesion synthesis (TLS), in which the replicative DNA polymerase is temporarily replaced by a specialized TLS polymerase capable of replicating across DNA lesions (Figure 1). The other is template switching (TS), which involves the switching of the stalled nascent DNA strand from the damaged template to undamaged sister strand for extension past the lesion (Figure 1). The initiation of TS is accomplished by the invasion of the sister chromatid by the stalled strand (Figure 1). The resulting D-loop is subsequently converted into a joint molecule called a sister chromatid junction (SCJ) after DNA synthesis using the intact sister strand as the template (Figure 1). SCJ can be disentangled to yield two duplex DNA strands via dissolution by the helicase/topoisomerase complex Sgs1/Top3/Rmi1 (STR) or resolution by the structure-specific nuclease Mus81/Mms4 complex (Figure 1). Note that although TLS is mechanistically simple, it is error-prone and largely accountable for mutagenesis. TS, on the other hand, is complex but error-free, and is therefore believed to be the preferred pathway. How cells choose a DTT pathway in response to genotoxin-induced DNA lesions is not clear. Gonzalez-Huici et al. found evidence suggesting that Hmo1 helps to channel MMS-induced DNA lesions to the TS pathway and facilitates the formation of the SCJ [17], but the underlying mechanism has yet to be resolved.

In this work, we found an extensive interplay of Hmo1 with components of the genome integrity network in the cellular response to MMS, which implicates Hmo1 in homology-directed DNA repair, replication-coupled chromatin assembly, and DNA damage checkpoint that participate in or impact DDT. Our results suggested that Hmo1 channels the TS intermediate SCJ to the Mus81/Mms4-mediated resolution pathway, which is parallel to the STR-mediated dissolution pathway. They also indicated that Hmo1 can contribute to DDT by modulating both the recycling of parental histones and the deposition of newly synthesized histones at the replication fork. We found evidence that Hmo1 and H2A.Z (Htz1 in yeast) have an antagonistic relationship in DDT, possibly due to their opposite impacts on DNA end resection. We also found that Hmo1 is required for DNA damage checkpoint signaling in response to MMS and for DNA negative supercoiling as a proxy of the chromatin structure. The CTD of Hmo1, and by inference its DNA bending activity, is dispensable for its ability to promote checkpoint signaling or DNA supercoiling. Moreover, we found the CTD of Hmo1 to be required for its modulation of MMS tolerance in cells lacking Htz1, but not in cells lacking any of several other DDT factors. In sum, our findings indicated that Hmo1 can contribute to or regulate DDT via multiple mechanisms in a genetic background-dependent manner.

## 2. Results

### 2.1. Distinct Functional Interactions of Hmo1 with DDT Factors Involved in the Formation and Processing/Removal of the SCJ Intermediate of Template Switching

**Figure 2:**
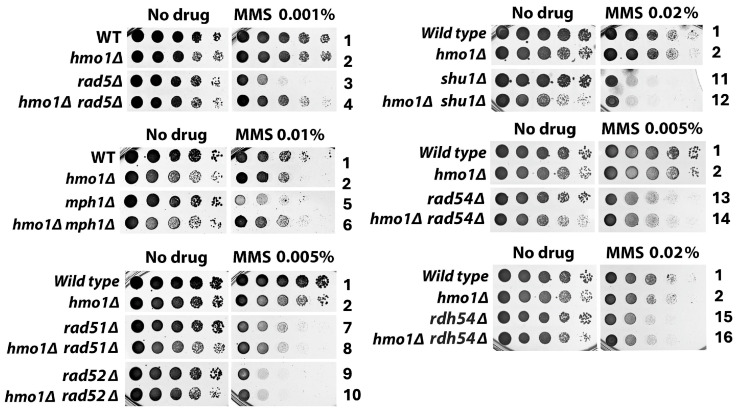
Functional interplay of Hmo1 with Rad5, Mph1, and homologous recombination factors in cellular tolerance to MMS. The growth phenotypes of the indicated strains (#1–16 in Table S1) in the presence or absence of MMS are shown. Cells of each strain were grown to saturation, and 10-fold serial dilutions of the culture were spotted on SC (synthetic complete) medium/plate with (MMS) or without (no drug) MMS. The plates were incubated for 3 days at 30 °C before their images were taken.

DDT, also known as post-replication repair, is intimately intertwined with DNA replication [20,21]. The sliding DNA clamp PCNA, a key replication factor, plays a central role in the selection of DDT pathways [21,22]. PCNA mono-ubiquitination promotes TLS whereas PCNA polyubiquitination by Rad5/Ubc13/Mms2 activates the TS pathway of DDT. Rad5 also has DNA helicase activity that promotes fork reversal that may lead to SCJ formation at a stalled replication fork [23,24]. Hmo1 has been implicated in the TS pathway of DDT, as the deletion of the *HMO1* gene (*hmo1Δ*) suppresses the hypersensitivity of *rad5Δ* mutant to MMS [17] (Figure 2, compare strains 3 and 4). Like Rad5, the Mph1 helicase also promotes fork reversal at a stalled replication fork [24]. We found that *hmo1Δ* also suppressed MMS-sensitivity of *mph1Δ* mutant (Figure 2, compare strains 5 and 6). These results are consistent with the notion that Hmo1 functions upstream of Rad5 and Mph1 to channel DNA lesions inflicted by MMS to the TS pathway.

Given that Hmo1 binds DNA with specificities for altered structures such as four-way junctions [25] that exist in TS intermediates such as SCJ (Figure 1), we wondered whether Hmo1 is also involved in TS processes downstream of Rad5 and Mph1. Accordingly, we examined the interplay of Hmo1 with TS factors responsible for the formation and processing/removal of SCJs in yeast tolerance to MMS. SCJ formation is mediated by core homologous recombination (HR) factors, including the Rad52 epistasis group of proteins and specialized HR factors such as the Shu complex (Shu1/Shu2/Psy3/Csm2) [26,27] (Figure 1). Rad51 recombinase promotes the invasion of sister chromatid by ssDNA, which is facilitated by Rad52 and the Shu complex [27,28,29]. Rad54 and Rdh54 aid in the homology search by the Rad51 presynaptic complex [30]. We found that *rad51Δ, rad52Δ*, *shu1Δ*, *rad54Δ* and *rdh54Δ* were all dominant over *hmo1Δ* in affecting cellular tolerance to MMS (Figure 2, compare strains 7 and 8, 9 and 10, 11 and 12, 13 and 14, and 15 and 16). As such, the *RDA51*, *RAD52*, *SHU1*, *RAD54* and *RDH54* genes were all epistatic to *HMO1* in cellular tolerance to MMS, suggesting a role for Hmo1 in the TS pathway downstream of HR factors that promote SCJ formation (Figure 1).

**Figure 3:**
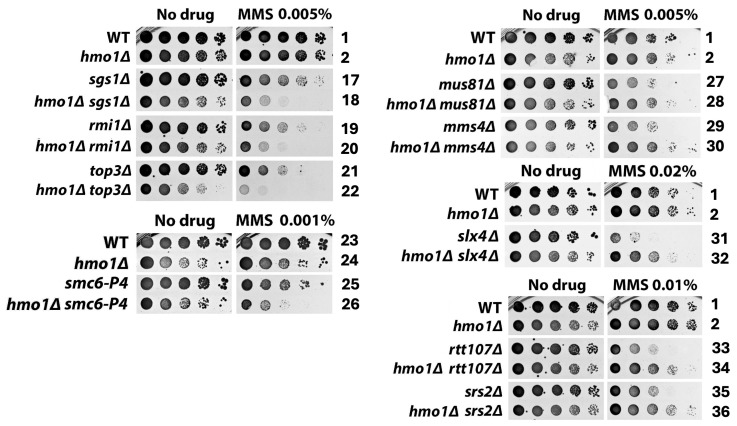
Hmo1 functions in the Mus81/Mms4-mediated resolution pathway independently of the Sgs1/Top3/Rmi1 (STR)-mediated dissolution pathway in DDT. The growth phenotypes of the indicated strains in the presence or absence of MMS are shown.

An SCJ can be converted to a hemicatenane that can be dissolved by the STR helicase/topoisomerase complex, or to a double Holliday junction (dHJ) that can be resolved by the structure-specific nuclease Mus81/Mms4 [26] (Figure 1). STR-mediated SCJ dissolution yields non-crossover (NCO) products, whereas Mus81/Mms4-mediated resolution may generate NCO or crossover (CO) products [26,31] (Figure 1). We found that *hmo1Δ* exacerbated the MMS sensitivity of the *sgs1Δ*, *top3Δ*, and *rmi1Δ* mutants (Figure 3, compare strains 17 and 18, 19 and 20, and 21 and 22). STR activity is stimulated by the Smc5/6-mediated SUMOylation of Sgs1, Top3 and Rmi1 [32]. We showed that *hmo1Δ* also exacerbated the MMS sensitivity of cells carrying the *smc6-P4* mutation, which disrupts the SUMO ligase function of Smc5/Smc6 (Figure 3, compare strains 25 and 26). These results indicated that Hmo1 functions separately from the Smc5/6-STR axis for SCJ dissolution in TS. On the other hand, *hmo1Δ* suppressed the MMS sensitivity of the *mus81Δ* and *mms4Δ* mutants (Figure 3, compare strains 27 and 28, and 29 and 30). Slx4 in complex with Rtt107 is involved in the activation of the nuclease activity of Mus81/Mms4, whereas Srs2 associates with Mus81/Mms4 and promotes its function in SCJ resolution [33,34]. We found *hmo1Δ* to also suppress the MMS sensitivity of the *slx4Δ, rtt107Δ*, and *srs2Δ* mutants (Figure 3). These results are consistent with the notion that Hmo1 acts upstream of Mus81/Mms4 and directs the SCJ to the resolution pathway of TS. Therefore, Hmo1 not only regulates the choice of the DTT pathway (TS vs. TLS) but also the choice of the SCJ processing mechanism (resolution vs. dissolution).

### 2.2. Hmo1 May Contribute to DDT by Functioning in DNA Replication-Coupled Chromatin Assembly

**Figure 4:**
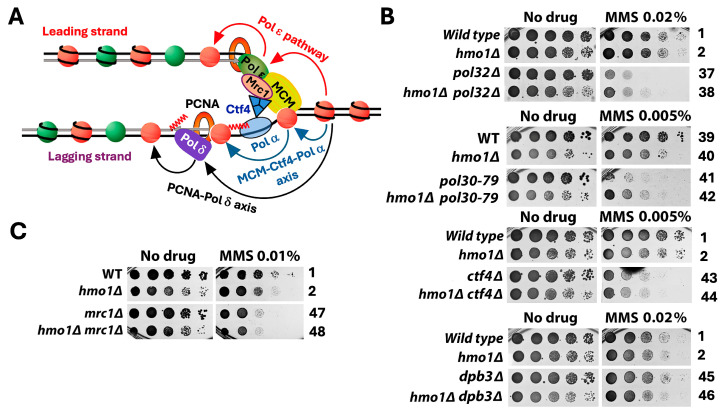
Hmo1 can contribute to DDT by modulating parental histone recycling at the replication fork. (**A**). Mechanisms of the strand-specific transfer of parental histones H3/H4 to the leading and lagging strands behind the replication fork. MCM, minichromosome maintenance protein complex, a DNA helicase. Wiggly red line, RNA primer. Green and red circles, nucleosomes bearing new and old H3/H4, respectively. The red, blue, and black curved arrows denote the pol ε pathway, MCM-Ctf4-Pol α axis, and PCNA-Pol δ axis of parental histone recycling, respectively. See the text for descriptions. (**B**,**C**). Growth phenotypes of the indicated strains in the presence or absence of MMS.

**Figure 5:**
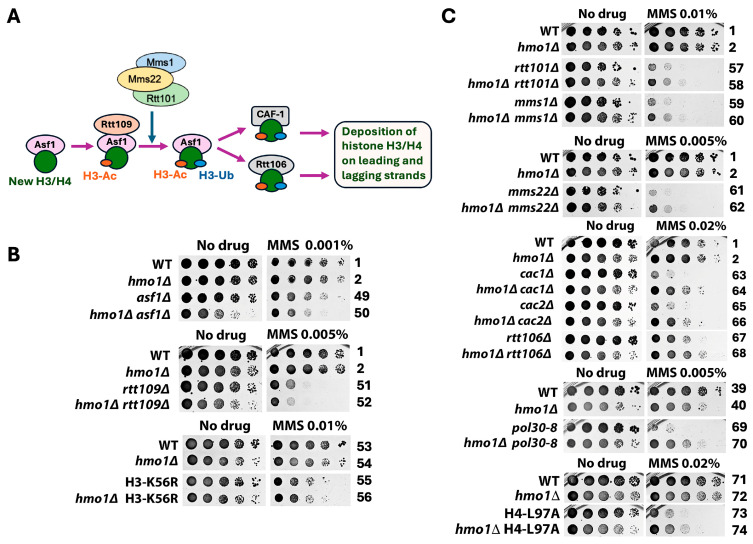
Hmo1 can contribute to DDT by modulating new histone deposition on the nascent strand during DNA replication. (**A**). Mechanism of the replication-coupled deposition of newly synthesized histones H3/H4 on DNA. H3-Ac, histone H3-K56 acetylation. H3-Ub, H3-K121,122,125 ubiquitination. CAF-1, Cac1/Cac2/Cac3 complex. See the text for descriptions. (**B**,**C**). Growth phenotypes of the indicated strains in the presence or absence of MMS.

DNA replication-coupled chromatin assembly plays a critical role in DDT [35]. Given that Hmo1 associates with chromatin throughout the cell cycle [13] and that Hmo1 can affect nucleosome structure and chromatin compaction [5,8,9,12], it is possible that Hmo1 contributes to DDT by impacting replication-coupled chromatin assembly. Nucleosome formation on nascent DNA is accomplished by the recycling of parental histones mediated by the replisome and the deposition of newly synthesized histones by chromatin assembly factors [36]. At the replication fork, the parental histone H3/H4 tetramer is displaced from the nucleosome and then distributed onto both leading and lagging strands by distinct strand-specific mechanisms. The transfer of histones H3/H4 to the leading strand is promoted by DNA polymerase Pol ε [37,38] and transfer to the lagging strand is governed by the PCNA-Pol δ and Mcm2-Ctf4-Pol α axes [39,40,41] (Figure 4A). Notably, the nonessential Dpb3/Dpb4 subunit of Pol ε and Pol32 subunit of Pol δ both bind histones H3/H4 directly [40,41]. Moreover, the replisome component Mrc1 also plays a role in the symmetric distribution of parental histones to sister chromatids [42,43,44] (Figure 4A). Newly made histones H3/H4 are deposited onto nascent DNA via a histone modification and nucleosome assembly pathway [36,45] (Figure 5A). In this pathway, the H3/H4 dimer associates with the histone chaperone Asf1, followed by the acetylation of lysine 56 of H3 (H3-K56-Ac) by histone acetylase Rtt109 and subsequent H3 ubiquitination (on K121, K122, and K125) by the Rtt101/Mms1/Mms22 ubiquitinase [35,36,46]. Histone H3 acetylation and ubiquitination facilitate the transfer of the H3/H4 dimer from Asf1 to chromatin assembly factors CAF-1 (Cac1/Cac2/Cac3 complex) and Rtt106 that deposit H3/H4 on the nascent strand (Figure 5A). Defects in parental histone recycling or new histone deposition make cells sensitive to genotoxins [35]. For instance, the *pol32Δ*, *ctf4Δ*, or *mrc1Δ* mutation sensitizes cells to MMS (Figure 4B,C, compare strains 37, 43, and 47 with 1), and the deletion of any component of the new histone deposition pathway or changing H3-K56 to non-acetylable arginine (H3-K56R) also makes cells sensitive to MMS (Figure 5B,C).

To examine whether Hmo1 impacts DDT by modulating replication-coupled chromatin assembly, we examined the interplay of Hmo1 with components of the parental histone transfer and new histone deposition pathways in cellular tolerance to MMS. We showed that *hmo1Δ* partially suppressed the MMS sensitivity of the *pol32Δ* mutant (Figure 4B, compare strains 37 and 38). Pol32 interacts with PCNA, and disrupting this interaction by the PCNA mutation *pol30-79* also sensitizes cells to MMS [47,48]. (Note that PCNA is a homotrimer of the Pol30 protein.) We found that *hmo1Δ* also suppressed the MMS sensitivity of the *pol30-79* mutant (Figure 4B, compare strains 41 and 42). These results placed Hmo1 upstream of Pol32 in the PCNA-Pol δ mediated pathway of parental histone H3/H4 transfer to the lagging strand. We found *CTF4* to be epistatic to *HMO1* in MMS tolerance (Figure 4B, compare strains 43 and 44), suggesting that Hmo1 functions downstream of Ctf4 in the MCM-Ctf4-Pol α-mediated parental histone transfer pathway. We noted that *dpb3Δ* had little effect on cellular tolerance to MMS (Figure 4B, compare strains 1 and 45), which is consistent with similar findings in prior studies [49]. The deletion of Hmo1 did not significantly affect the survival of the *dpb3Δ* mutant in the presence of MMS (Figure 4B, compare strains 45 and 46). In addition, we found *MRC1* to be epistatic to *HMO1* in MMS tolerance (Figure 4C, compare strains 47 and 48), suggesting that Hmo1 functions downstream of Mrc1 in regulating parental histone transfer. These results suggest that Hmo1 functions in parental histone recycling, especially lagging strand-specific histone transfer that seems to impact DDT more than leading strand-specific histone transfer.

We observed an additive/synergistic effect of *hmo1Δ* with *asf1Δ* or *rtt109Δ* on unperturbed cell growth (Figure 5B, “No drug” panel, compare strain 50 with strains 1, 2, and 49; compare strain 52 with strains 1, 2, and 51), indicating that Hmo1 contributes to cell growth separately from Asf1 and Rtt109. The survival/growth of the *asf1Δ* and *rtt109Δ* mutants in the presence of MMS was reduced by *hmo1Δ* to a degree similar to the *hmo1Δ*-induced growth reduction in the absence of MMS (Figure 5B). As such, *hmo1Δ* did not significantly affect the MMS sensitivity of *asf1Δ* or *rtt109Δ* mutant. Likewise, *hmo1Δ* did not affect the MMS sensitivity of the H3-K56R mutant (Figure 5B). Therefore, *ASF1* and *RTT109* are epistatic to *HMO1* in MMS tolerance, suggesting that Hmo1 functions in the new histone deposition pathway downstream of Asf1/Rtt109-mediated H3-K56 acetylation. To further test this notion, we examined the interplay of Hmo1 with Rtt101/Mms1/Mms22, CAF-1, and Rtt106 that function downstream of Asf1/Rtt109 (Figure 5A). As shown in Figure 5C, *hmo1Δ* suppressed the MMS sensitivity of each of the *rtt101Δ*, *mms1Δ*, *mms22Δ*, *cac1Δ*, *cac2Δ* and *rtt106Δ* mutants. CAF-1 recruitment to replicating DNA is mediated by the Cac1 interaction with PCNA, and disrupting this interaction by *pol30-8* mutation results in MMS sensitivity [50]. We showed that *hmo1Δ* also suppressed the MMS sensitivity of the *pol30-8* mutant (Figure 5C). The histone H4-L97A mutation hinders the deposition of histone H3/H4 dimer onto DNA by CAF-1 or Rtt106 [51]. We found *hmo1Δ* to also suppress the MMS sensitivity of cells bearing the histone H4-L97A mutation (Figure 5C). These results suggest that Hmo1 can contribute to DTT by functioning downstream of Asf1/Rtt109 and upstream of Rtt101/Mms1/Mms22, CAF-1, and Rtt106 in the new histone deposition pathway.

### 2.3. Hmo1 Antagonizes the Function of the Histone H2A Variant H2A.Z (Htz1 in Yeast) in DDT

**Figure 6:**
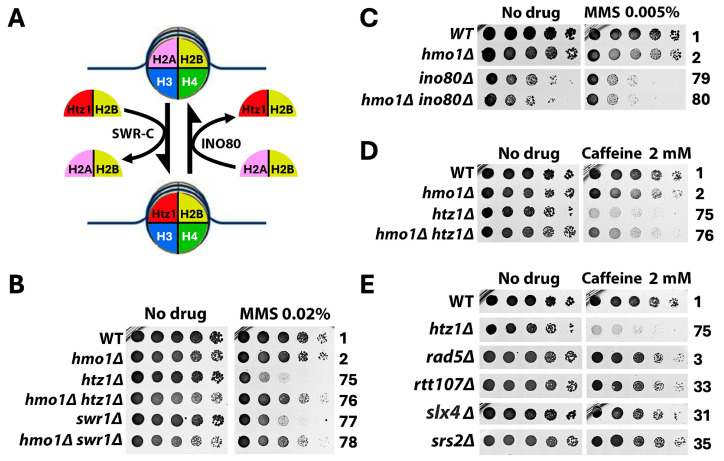
Hmo1 antagonizes the function of the histone H2A variant H2A.Z (Htz1) in yeast tolerance to MMS. (**A**). Mechanisms of the incorporation of Htz1 into chromatin and its removal from chromatin. SWR-C catalyzes the replacement of the H2A/H2B dimer in the nucleosome with the Htz1/H2B dimer, whereas INO80 catalyzes the reverse reaction. (**B**,**C**). Growth phenotypes of the indicated strains in the presence or absence of MMS. (**D**,**E**). Growth phenotypes of the indicated strains on medium with or without caffeine.

The highly conserved histone H2A variant H2A.Z (Htz1 in yeast) plays multiple roles in genome maintenance and function [52,53]. Histone H2A.Z deposition and removal are mediated by the chromatin remodelers SWR-C and INO80, respectively [54,55,56] (Figure 6A). There is evidence implicating H2A.Z in the cellular response to replicative stress [57]. Yeast Htz1 deposition and retention in chromatin were found to prevent replisome uncoupling at stalled replication forks, avoiding fork collapse [57]. We examined the interplay of Hmo1 with Htz1 and Swr1 (catalytic subunit of SWR-C) and found that *hmo1Δ* suppressed the MMS sensitivity of both the *htz1Δ* and *swr1Δ* mutants (Figure 6B, compare strains 75 and 76, and strains 77 and 78). A previous survey of the genome-wide distribution of Hmo1 found an inverse correlation between Hmo1 association and Htz1 binding in the yeast genome [13], suggesting a competitive/antagonistic relationship between Hmo1 and Htz1 in their association with the genome and by inference their functions in the genome. In support of this notion, we found that the *INO80* gene encoding Ino80, the catalytic subunit of INO80 complex, was epistatic to *HMO1* in MMS tolerance (Figure 6C, compare strains 79 and 80), implicating Hmo1 in the INO80-mediated Htz1 removal pathway.

Consistent with an antagonistic relationship between Htz1 and Hmo1, they have been shown to have opposing effects on DNA resection in DSB repair, with Htz1 promoting and Hmo1 inhibiting resection [16,58]. The *htz1Δ* mutant is hypersensitive to caffeine that inhibits DNA resection [54,59], likely because the *htz1Δ* mutation and caffeine together reduce DNA resection to a level that is too low for the effective repair of spontaneous DNA damage generated during cell proliferation. We showed that the hypersensitivity of the *htz1Δ* mutant to caffeine was partially suppressed by *hmo1Δ* (Figure 6D, compare strains 75 and 76), possibility due to an increase in DNA resection as a result of removing the inhibitory effect of Hmo1. It is possible that the suppression of the MMS sensitivity of *htz1Δ* mutant by *hmo1Δ* (Figure 6B) also reflects an antagonistic relationship of Htz1 and Hmo1 in impacting DNA resection at the stalled replication fork during DDT.

### 2.4. Genetic Background-Dependent Differential Requirement of the CTD of Hmo1 for Its Function in DDT

**Figure 7:**
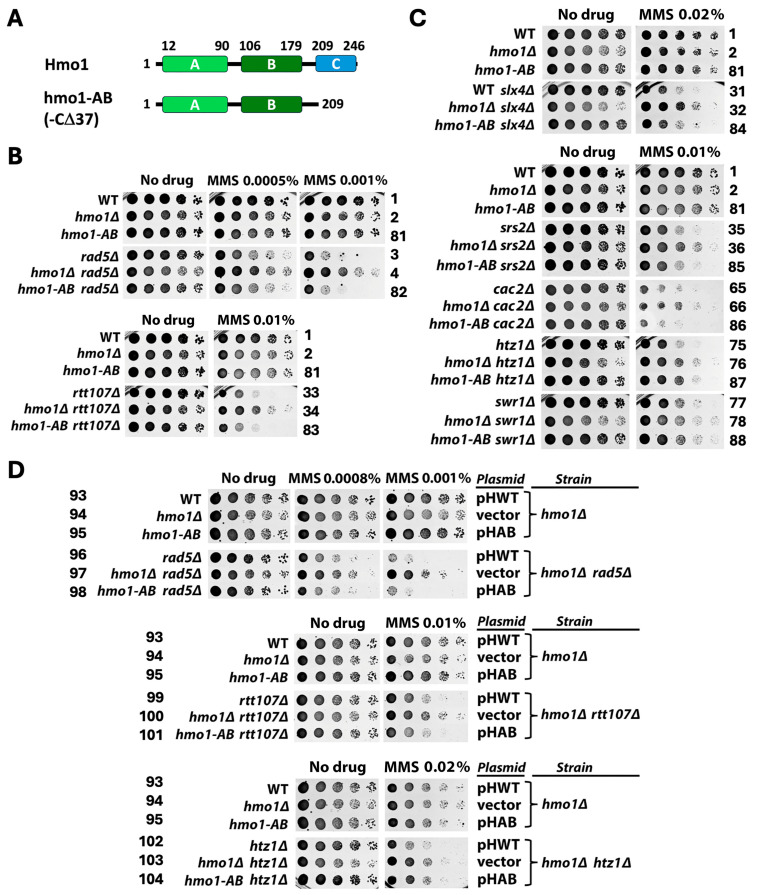
The CTD of Hmo1 contributes to MMS tolerance in a genetic background-dependent manner. (**A**). Domain structure of Hmo1. Top panel, domains (HMGB motifs) A and B, and the basic CTD of Hmo1. Bottom panel, the hmo1-AB allele (Hmo1 deleted for the CTD). (**B**–**D**). Growth phenotypes of the indicated strains in the presence or absence of MMS. The plasmids pHWT (pRS416-*HMO1*), vector (pRS416), and pHAB (pRS416-*hmo1-AB*) and their host strains are indicated on the right of the plate images in (**D**). The plasmids pHWT and pHAB have been described before [62].

Hmo1 has two HMGB motifs (referred to as domains A and B hereafter) and a basic CTD spanning residues 209–246 [6,7] (Figure 7A). Domain A and, to a lesser extent, domain B bind DNA with a specificity for altered DNA structures, such as four-way junctions [25]. Hmo1 can also bend DNA, which requires its CTD [60,61]. Deletion of the CTD does not affect Hmo1 stability or its DNA binding ability [25,60,61]. Chromatin in cells bearing Hmo1 deleted for CTD, referred to as hmo1-AB, was hypersensitive to nuclease digestion, similar to chromatin in cells deleted for Hmo1, suggesting that the CTD is required for the ability of Hmo1 to compact chromatin [12]. There is also evidence suggesting that the deletion of Hmo1 or its CTD results in a more dynamic chromatin environment at DSBs [15]. However, whereas Hmo1 deletion results in a salient growth defect in yeast, cells expressing hmo1-AB exhibit no growth defect [60,61]. As such, the function of Hmo1 required for normal cell proliferation does not reside within its CTD.

To test if CTD of Hmo1 is required for its function in DDT, we examined if the *hmo1-AB* mutation resembles *hmo1Δ* in affecting MMS tolerance. We first tested if *hmo1-AB* could suppress the MMS sensitivity of the *rad5Δ* mutant, similar to *hmo1Δ*. As shown in Figure 7B, unlike *hmo1Δ*, *hmo1-AB* did not suppress the MMS sensitivity of the *rad5Δ* mutant (compare strains 3 and 82). We found that *hmo1-AB* also failed to suppress the MMS sensitivity of the *rtt107Δ*, *slx4Δ*, *srs2Δ* or cac2*Δ* mutant (Figure 7B,C). However, like *hmo1Δ*, *hmo1-AB* suppressed the MMS sensitivity of the *htz1Δ* and *swr1Δ* mutants (Figure 7C). These results indicate that the CTD of Hmo1 is dispensable for its function in DDT in cells lacking Rad5, Rtt107, Slx4, Srs2, or Cac2, but is necessary for Hmo1 function in cells lacking Htz1 or Swr1. Note that in the above experiment, the *hmo1-AB* mutation was introduced into the endogenous *HMO1* locus of the host. We were able to reproduce the results demonstrating the ability of *hmo1-AB* to suppress *htz1Δ* but not *rad5Δ* or *rtt107Δ* by expressing the *hmo1-AB* allele carried on a plasmid (Figure 7D). The above results indicate that the CTD of Hmo1 may or may not be required for Hmo1 function in DDT, depending on the genetic background of the host.

Our finding that *hmo1-AB* did not suppress the MMS sensitivity of the *rad5Δ* mutant is not consistent with an earlier report showing that the deletion of C-terminal 64 or 22 residues of Hmo1 (hmo1-*Δ*64 or hmo1-*Δ*22) partially suppressed the MMS sensitivity of the *rad5Δ* mutant [17]. The *hmo1-AB* allele we used here has been characterized in several prior investigations on Hmo1 functions [15,16,61,62]. It is deleted for the C-terminal 37 residues (from 209 to 246) of Hmo1 (Figure 7A), which is more than the deletion of the hmo1-*Δ*22 allele but less than the deletion of the hmo1-*Δ*64 allele used in the work of Gonzalez-Huici et al. [17]. Therefore, the discrepancy between our result and that of Gonzalez-Huici et al. is unlikely due to the difference in the size of the truncation of the Hmo1 alleles. Note that the strains we used here were derived from BY4741 (Table S1) whereas those used by Gonzalez-Huici et al. were W303 derivatives [17]. It is possible that the discrepancy regarding the role of Hmo1-CTD in MMS tolerance in the *rad5Δ* mutant stemmed from the difference in the genetic background between BY4741 and W303 [63].

### 2.5. Hmo1 Promotes DNA Damage Checkpoint Signaling Induced by MMS

**Figure 8:**
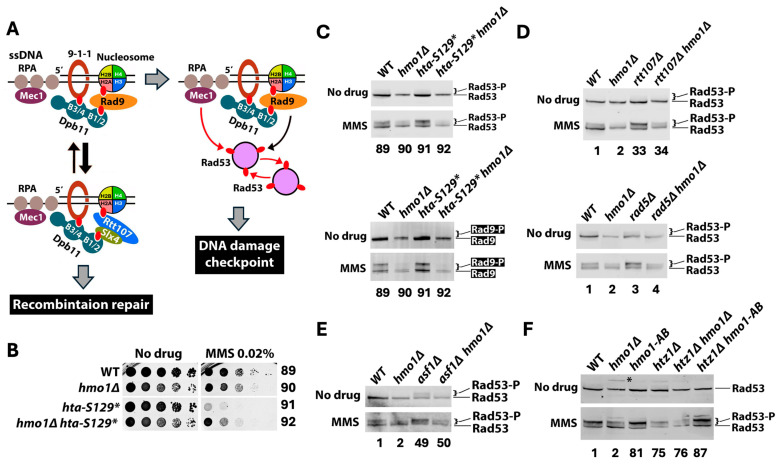
Hmo1 promotes DNA damage checkpoint signaling induced by MMS. (**A**) Model of the dynamic balance between DNA damage checkpoint signaling and DNA recombination repair achieved by the competition between Rad9 and Slx4/Rtt107 for binding γH2A and Dpb11 at damaged chromatin. The DNA damage clamp 9-1-1 binds the junction between dsDNA and 5′ ssDNA of a ssDNA gap or resected DSB end. Mec1 bound to RPA-ssDNA phosphorylates 9-1-1 and H2A (yielding γH2A). Phosphorylated 9-1-1 recruits the scaffold protein Dpb11 that contains four BRCT domains (designed B1 to B4). B3 and B4 (B3/4) of Dpb11 bind phosphorylated 9-1-1, whereas B1 and B2 (B1/2) can bind phosphorylated Rad9 or phosphorylated Slx4. Both Rad9 and Rtt107 can bind γH2A. Filled red oval, phosphate group. Red curved arrow, phosphorylation. Black curved arrow, activation of Rad53 by Rad9. See the text for more descriptions. (**B**) Growth phenotypes of strains 89–92 (Table S1) in the presence or absence of MMS. (**C**–**F**). Results from Western blot analyses of Rad53 and Rad9-HA from the indicated strains with (MMS) or without (No drug) MMS treatment. Log-phase cells were treated with (MMS) or without (no drug) MMS at 0.02% for 90 min. Protein extracts from each cell culture was subjected to SDS-PAGE and Western blotting, followed by the detection of Rad53 and Rad9-HA with Rad53 and HA antibodies, respectively. Rad53-P, phosphorylated Rad53 protein; Rad9-P, phosphorylated Rad9-HA protein. The asterisk denotes a protein/band that cross-reacted with the Rad53 antibody.

MMS induces the DNA damage checkpoint (DDC) in S phase [64,65]. DDC is initiated by Mec1 kinase bound to the ssDNA binding protein RFA coating ssDNA gaps and facilitated/amplified by the Rad9 mediator at stalled replication forks [66]. Rad9 is activated by Mec1-mediated phosphorylation, and phosphorylated Rad9 (Rad9-P) then binds and activates the checkpoint effector Rad53 kinase, which is autophosphorylated and phosphorylated by Mec1 [66]. Rad9 is recruited to damaged chromatin by Dpb11 and γH2A (histone H2A phosphorylated at S129, which is equivalent to γH2AX in higher eukaryotes) [66] (Figure 8A). Notably, the DNA repair scaffold Slx4/Rtt107 is also recruited to DNA lesions by Dpb11 and γH2A [67,68,69] (Figure 8A). The competition between Rad9 and Slx4/Rtt107 for binding γH2A and Dpb11 is thought to yield a dynamic balance between Rad9-mediated checkpoint signaling and Slx4/Rtt107-promoted DNA recombination repair [70] (Figure 8A). Along this line, *rtt107Δ* or *slx4Δ* results in enhanced Rad9 recruitment and heightened checkpoint signaling, as reflected by increased Rad9 phosphorylation (Rad9-P) and Rad53-P in response to MMS [70,71]. We have recently found that blocking histone H2A-S129 phosphorylation by the hta-S129* mutation also increases the levels of Rad9-P and Rad53-P induced by MMS [71]. (Note the hta-S129* allele of histone H2A is deleted for the C-terminal four residues including S129.) This led to the notion that Slx4/Rtt107 normally outcompetes Rad9 for binding γH2A at DNA lesions induced by MMS (Figure 8A), and, consequently, γH2A negatively regulates DDC in response to MMS by hindering Rad9 recruitment [71].

The *slx4Δ*, *rtt107Δ*, and hta-S129* mutants were all hypersensitive to MMS (Figure 3 and Figure 8B), which could be caused by heightened checkpoint and/or reduced recombination repair in them. We found that *hmo1Δ* suppressed the MMS sensitivity of the htaS-129* mutant, similarly to that of the *slx4Δ* or *rtt107Δ* mutant (Figure 3 and Figure 8B). We reasoned that if excessive checkpoint activation in these mutants was the cause of MMS sensitivity, then suppression of MMS sensitivity by *hmo1Δ* should be accompanied by a reduction in checkpoint signaling. Consistent with this notion, we found that the level of MMS-induced Rad53-P (relative to unphosphorylated Rad53) in the *rtt107Δ hmo1Δ* or hta-S129* hmo1Δ double mutant was significantly lower than that in the *rtt107Δ* or hta-S129* single mutant, respectively (Figure 8C, top, compare strains 91 and 92; Figure 8D, compare strains 33 and 34). Moreover, the level of MMS-induced Rad9-P in hta-S129* hmo1Δ double mutants was markedly lower than that in the hta-S129* single mutant (Figure 8C, bottom panel, compare strains 91 and 92). As such, in the absence of Hmo1, *rtt107Δ* or hta-S129* was no longer able to induce the hyperactivation of DDC signaling. Notably, we also found that MMS-induced Rad53-P and Rad9-P levels in the *hmo1Δ* mutant were markedly lower than those in *HMO1* wildtype cells (Figure 8C, compare strains 89 and 90), indicating that Hmo1 has a general role of promoting Rad9-mediated checkpoint activation in response to MMS.

Taken together, the above results are consistent with the notion that excessive DDC activation induced by MMS would reduce the survivability of the host, which is subject to suppression by alleviating DDC (e.g., via deleting Hmo1). The *rad5Δ* and *asf1Δ* mutations also caused enhanced DDC, as reflected by increased MMS-induced Rad53-P levels (Figure 8D, compare strains 1 and 3; Figure 8E, compare strains 1 and 49). We found that *hmo1Δ* reduced MMS-induced Rad53-P levels in the *rad5Δ* mutant (Figure 8D, compare strains 3 and 4) and suppressed its MMS sensitivity (Figure 2). However, *hmo1Δ* reduced the MMS-induced change in Rad53-P level in the *asf1Δ* mutant (Figure 8E, compare strains 49 and 50) without suppressing its MMS sensitivity (Figure 5B). Therefore, whether elevated DDC causes MMS sensitivity may be dependent on the genetic background of the host.

To gain insights into how Hmo1 promotes DDC, we tested if the CTD of Hmo1 is required for this function. We found that unlike *hmo1Δ*, the *hmo1-AB* mutation did not reduce the level of Rad53-P induced by MMS (Figure 8F, compare strain 81 with strains 1 and 2). Therefore, the ability of Hmo1 to promote DDC does not depend on its CTD and by inference its DNA bending activity. While *hmo1-AB* suppressed the MMS sensitivity of the *htz1Δ* mutant similar to *hmo1Δ* (Figure 7C), it did not reduce Rad53-P levels in the *htz1Δ* mutant as did *hmo1Δ* (Figure 8F). This indicates that Hmo1’s function in DDT in the *htz1Δ* mutant is independent of its ability to promote checkpoint signaling.

### 2.6. Hmo1 Regulates DDT Independently of Its Promotion of Negative DNA Supercoiling as a Proxy of the Chromatin Structure

**Figure 9:**
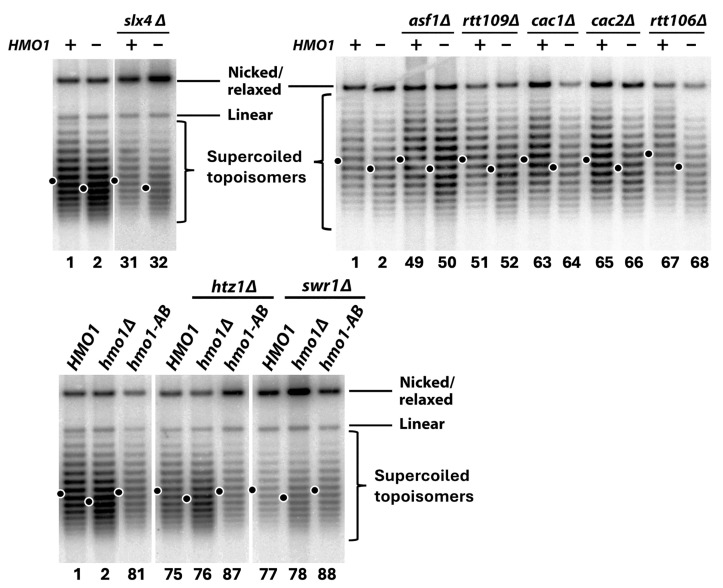
Hmo1 promotes negative DNA supercoiling as a proxy of the chromatin structure. DNA from cells of each indicated strain was subjected to agarose gel electrophoresis in the presence of 12 μg/mL chloroquine. Each sample (lane) is labeled with the host strain number (Table S1) at the bottom. The 2μ plasmid was detected via Southern hybridization with a 2μ plasmid-specific probe. Under the conditions used here, more negatively supercoiled topoisomers migrate more slowly. The positions of nicked/relaxed and linear plasmids are indicated. The center of distribution of 2μ topoisomers from each strain is denoted by a filled circle on the left of the lane.

Hmo1 has been shown to alter the nucleosome conformation in vitro and promote chromatin compaction in vivo [5,8,9,12]. Since nucleosome formation introduces negative supercoiling into DNA, the level of DNA negative supercoiling can be used as an indicator of chromatin formation/structure [72,73]. We found that *hmo1Δ* reduced the negative supercoiling of the 2-micron plasmid (2μ) in yeast by a linking number difference (ΔLk) of one (Figure 9, compare strains 1 and 2), suggesting that Hmo1 promotes nucleosome/chromatin formation and/or maintenance. We wondered if the role of Hmo1 in DNA negative supercoiling as a proxy of the chromatin structure is linked to its function in DDT. To address this question, we examined if the interplay of Hmo1 with DDT factors, especially those known to modulate the structure and/or replication of chromatin, such as Asf1/Rtt109 and CAF-1, is dependent on the negative DNA supercoiling promoted by Hmo1.

We found that none of the *slx4Δ*, *asf1Δ*, *rtt109Δ*, *cac1Δ*, *cac2Δ*, *rtt106Δ*, *htz1Δ*, and *swr1Δ* mutations affected 2μ supercoiling (Figure 9, compare strain 1 with strains 31, 49, 51, 63, 65, 67, 75, and 77). As such, the MMS sensitivity caused by these mutations was likely not due to a change in the general chromatin structure. The deletion of *HMO1* from any of these mutants reduced 2μ supercoiling by a ΔLk of one, similarly to the otherwise wildtype strain (Figure 9, e.g., compare strains 31 and 32). Note that *hmo1Δ* suppressed the MMS sensitivity of the *slx4Δ*, *cac1Δ*, *cac2Δ*, *rtt106Δ*, and *htz1Δ* mutants (Figure 3, Figure 5C and Figure 6B) but did not affect the MMS sensitivity of the *asf1Δ* or *rtt109Δ* mutant (Figure 5B). These results suggest that there is not a simple link between how Hmo1 affects MMS tolerance and Hmo1-dependent negative DNA supercoiling as a proxy of chromatin structure.

We wondered if DNA bending mediated by Hmo1-CTD is required for the function of Hmo1 in promoting DNA supercoiling. To address this question, we tested the effect of *hmo1-AB* mutation on 2μ supercoiling. We found that, unlike *hmo1Δ*, the *hmo1-AB* mutation did not affect 2μ supercoiling (Figure 9, bottom panel, compare strain 81 with strains 1 and 2), demonstrating that the CTD of Hmo1 is dispensable for its ability to promote DNA supercoiling. We also showed that the *hmo1-AB* mutation did not affect 2μ supercoiling in the *htz1Δ* or *swr1Δ* mutant (Figure 9, bottom panel, compare strain 87 with strains 75 and 76, and strain 88 with strains 77 and 78). That both *hmo1Δ* and *hmo1-AB* mutations suppressed the MMS sensitivity of the *htz1Δ* or *swr1Δ* mutant (Figure 7C) but only *hmo1Δ* reduced DNA supercoiling in these mutants (Figure 9) supports the notion Hmo1 can regulate DDT independently of its impact on DNA supercoiling as a proxy of the chromatin structure.

## 3. Discussion

### 3.1. Hmo1 May Direct Persistent SCJs to the Mus81/Mms4 Nuclease-Mediated Resolution Pathway in Template Switching

We found evidence implicating Hmo1 in the SCJ resolution pathway mediated by the Slx4-Mus81/Mms4 axis instead of the parallel SCJ dissolution pathway mediated by the Smc5/6-STR axis (Figure 1 and Figure 3). The suppressive effect of *hmo1Δ* on the MMS sensitivity of the *mus81Δ*, *mms4Δ*, and *slx4Δ* mutants can be explained by assuming that Hmo1 channels SCJs induced by MMS to the Mus81/Mms4-dependent resolution pathway. It is noteworthy that both the STR dissolvase and Mus81/mms4 resolvase are regulated by the cell cycle. STR is subject to S-phase specific Smc5/6-mediated SUMOylation that is required for efficient STR function [74], whereas Mus81/mms4 activation depends on the phosphorylation of Mms4 and the formation of a multiprotein complex containing Mus81/Mms4 and Slx4/Dpb11 in G2/M phase [33,75,76]. As such, it is believed that STR acts in S phase to dissolve SCJs, thus preventing unscheduled recombination during DNA replication that may lead to DNA rearrangement, while Mus81/Mms4 acts in G2/M phase to resolve SCJs that escape STR action [76]. We envision that Hmo1 may act upstream of the Mus81/Mms4 in SCJ resolution pathway by promoting the formation and/or stability of dHJs. Hmo1 may aid in the conversion of SCJs to dHJs by facilitating the reannealing of parental strands in SCJs (Figure 1). Alternatively, or in addition, Hmo1 may bind and stabilize preformed dHJs. In any case, when Mus81/Mms4 are absent, unresolved dHJs accumulate and become toxic, which can be alleviated by an Hmo1 deletion that reduces the abundance of dHJs destinated to be processed by Mus81/Mms4. Given that the error-prone TLS pathway of DDT also functions in G2/M phase [21], Hmo1 mediated channeling of SCJs to the resolution pathway in G2/M phase may serve to divert DNA lesions from TLS, thereby reducing the rate of mutagenesis.

### 3.2. Hmo1 May Regulate DDT by Modulating Replication-Coupled Chromatin Assembly

DDT is intimately intertwined with DNA replication and chromatin packaging. As a sequence-nonspecific DNA binding protein that preferentially binds DNA with altered conformations, Hmo1 may regulate the formation, stability, and/or processing of special DNA structures generated during ordinary DNA replication or replication stress. These structures include ssDNA gaps, D-loops, SCJs, and dHJs (Figure 1). In addition, since Hmo1 can bind and alter the nucleosome structure [8,9], it may modulate nucleosome assembly that is tightly coupled with ongoing DNA replication [77]. It is reasonable to posit that the ability of Hmo1 to bind altered DNA structures and its ability to modulate nucleosome assembly/structure mutually influence each other. The interplay of Hmo1 with the replication-coupled chromatin assembly machinery in MMS tolerance we observed (Figure 4 and Figure 5) suggests that Hmo1 may modulate DDT by functioning in both parental histone transfer and new histone deposition in the wake of the replication fork (Figure 4A and Figure 5A). Hmo1 likely acts upstream of Pol32 in the PCNA-Pol δ axis and downstream of Ctf4 in the MCM-Ctf4-Pol α axis of lagging strand-specific parental histone transfer pathways, as well as upstream of Rtt101/Mms22/Mms1 in the new histone deposition pathway. In doing so, Hmo1 likely promotes the creation of a state of nascent chromatin at a stalled replication fork that renders DNA lesions or ssDNA gaps more accessible to TS factors such as Rad5 and Mph1 than the TLS machinery, thereby channeling DNA lesions to the TS pathway (Figure 1). Similarly, Hmo1 may also aids in the establishment of a chromatin environment at SCJs that is conducive to its conversion to dHJs destinated to resolution by the Mus81/Mms4 nucleases (Figure 1).

### 3.3. Hmo1 Counteracts the Function of Histone H2A Variant H2A.Z (Htz1) in DDT

Our finding that *hmo1Δ* suppresses the hypersensitivity of *htz1Δ* mutant to caffeine, an inhibitor of DNA resection (Figure 6C), is in line with the antagonistic relationship of Htz1 and Hmo1 in DNA resection involved in DSB repair [15,58]. Htz1 promotes DSB repair likely because it makes the nucleosome/chromatin structure more accessible [58]. On the other hand, Hmo1 inhibits DSB end resection, possibly by stabilizing chromatin [15]. Based on the inverse correlation between Htz1 and Hmo1 in genome association [13], we propose that Htz1 counteracts Hmo1 binding to DNA/chromatin, thereby alleviating its inhibition of DSB repair. Consistent with this notion, it has been shown that Htz1 is recruited to the vicinity of DSBs and Hmo1 is evicted from it during DSB repair [15,58]. There is evidence implicating DNA resection in the formation/expansion of ssDNA gaps at stalled replication forks [78,79,80]. It is tempting to assume that, similar to DNA resection in DSB repair, DNA resection involved in DDT is also subject to positive regulation by Htz1 and negative regulation by Hmo1. This hypothesis can explain the suppression of the MMS sensitivity of the *htz1Δ* mutant by *hmo1Δ* (Figure 6B).

### 3.4. The Contribution of the CTD of Hmo1 to DDT Is Dependent on the Genetic Background of the Host

The molecular mechanisms underlying Hmo1’s functions in DDT have yet to be elucidated. Hmo1 has both HMGB-like characteristics and linker histone-like characteristics [6,7]. It is not clear how any of these characteristics may contribute to Hmo1 function in DDT. The lysine-rich basic CTD of Hmo1 resembles that of canonical linker histones and is believed to be the structural basis of Hmo1’s linker histone-like characteristics [6,7]. The CTD of Hmo1 is required for its DNA bending activity but is dispensable for its DNA-binding activity [15,25,60,61]. The CTD and by inference DNA bending are required for Hmo1’s abilities to make the yeast genome resistant to nuclease digestion possibly due to chromatin compaction [12] and to stabilize chromatin (promoting a less dynamic chromatin environment) [15]. However, we found that the CTD of Hmo1 is not required for its function in promoting the negative supercoiling of DNA (Figure 9), which is an indicator of the formation of nucleosomes/primary chromatin [72,73]. Therefore, Hmo1 seems to contribute to the formation of primary chromatin and chromatin compaction via distinct CTD-independent and CTD-dependent mechanisms, respectively. The CTD of Hmo1 has recently been found to be necessary for its interaction with chromatin remodeling complexes [11], which raises the possibility that Hmo1 promotes chromatin compaction by recruiting chromatin remodeling complexes.

That *hmo1-AB* phenocopied *hmo1Δ* in suppressing the MMS sensitivity of *htz1Δ* or *swr1Δ* mutant (Figure 7C) pointed to a role for the CTD of Hmo1 in DDT. However, unlike *hmo1Δ*, *hmo1-AB* failed to suppress the MMS sensitivity of the *rad5Δ, rtt107Δ, slx4Δ, srs2Δ* or cac2*Δ* mutant (Figure 7B,C), demonstrating that the CTD is dispensable for Hmo1 function in these mutants. These results indicate that Hmo1 may function in DDT via CTD-dependent or CTD-independent mechanisms, depending on the genetic background of the host. The *htz1Δ* mutant is hypersensitive to caffeine, which inhibits DNA resection [54,59], and is suppressed by *hmo1Δ* (Figure 6B). On the other hand, we found that none of the *rad5Δ*, *rtt107Δ*, *slx4Δ*, or *srs2Δ* mutants was sensitive to caffeine (Figure 6E). It is possible that the unique circumstance of the *htz1Δ* mutant (e.g., reduced DNA resection capability) enables or necessitates a CTD-dependent function of Hmo1 (e.g., inhibition of DNA resection) in DDT. Such a CTD-dependent Hmo1 function may not take place or be required for DDT in the *rad5Δ*, *rtt107Δ*, *slx4Δ*, or *srs2Δ* mutant (in which DNA resection is not reduced, as in the *htz1Δ* mutant). How and under what circumstances the CTD of Hmo1 contributes to its function in DDT await further investigations.

### 3.5. Hmo1 Promotes DNA Damage Checkpoint Signaling

Our finding that *hmo1Δ* reduced MMS-induced Rad53-P and Rad9-P in yeast (Figure 8C) demonstrated a role for Hmo1 in promoting DNA damage checkpoint signaling in response to replication stress. Hmo1 may help create a local chromatin environment that is favorable for the functions of the DDC kinase Mec1 and/or mediator Rad9. Mec1 is recruited to ssDNA by the ssDNA-binding protein RPA [66] (Figure 8A). As RPA-bound sites in the genome overlap those of Hmo1-associated sites in the yeast genome upon MMS treatment [17], it is possible that Hmo1 contributes to the stability of RPA-covered ssDNA regions at stalled replication forks, thereby facilitating Mec1 recruitment. Rad9 is recruited to damaged chromatin in part by associating with γH2A [66] (Figure 8A). Hmo1 may aids in Rad9 recruitment by altering the structure of γH2A-bearing nucleosomes in a manner that increases the affinity of γH2A for Rad9, instead of its competitive inhibitor Rtt107 (Figure 8A). Notably, we found that the CTD of Hmo1 is not required for its role in promoting DDC in response to MMS (Figure 8F). We found that the ability of Hmo1 to promote DDC signaling is not required for its function in DDT in the *htz1Δ* mutant (Figure 8F). A decrease in DDC signaling caused by *hmo1Δ* is correlated with an improvement of MMS tolerance in mutants such as *rtt107Δ* but not in the *asf1Δ* mutant (Figure 8D,E). Therefore, Hmo1-promoted DDC signaling may be irrelevant to DDT or may only be involved in DDT in certain genetic backgrounds.

## 4. Materials and Methods

### 4.1. Yeast Strains

The yeast strains used in this work are listed in Table S1. Yeast mutants were made by replacing the open reading frames (ORFs) of the genes of interest with the selectable marker gene *NatMX* or *URA3*. This was achieved by gene replacement in which a PCR product consisting of a marker gene flanked by sequences homologous to the target gene was introduced/transformed into the parental strain. Homologous recombination between the PCR product and the target gene in the genome would then result in the replacement of the ORF of the target gene with the marker gene. The relevant genotype of each mutant made was verified by Southern blotting or PCR. Strains W1588-4C and T382-P4 were obtained from Dr. Xiaolan Zhao (Memorial Sloan Kettering Cancer Center); PY39, PY39-8, and PY30-79 from Dr. Peter Burgers (Washington University); and QY364 and QY375 from Dr. Stephen Kron (University of Chicago). The plasmids pHWT (pRS416-*HMO1*) and pHAB (pRS416-*hmo1-AB*) were obtained from Dr. Anne Grove (Louisiana State University). Synthetic complete (SC) medium was used for growing yeast cells.

### 4.2. Examination of the Yeast Growth Phenotype

Cells of a yeast strain were grown in synthetic complete (SC) liquid medium overnight to saturation. Ten-fold serial dilutions of the culture were spotted on test plates (SC solid medium) with or without MMS or caffeine. The plates were incubated at 30 °C for 3 days before their images were taken.

### 4.3. SDS-PAGE and Western Blotting

Protein extracts from yeast cells were obtained by TCA extraction [81]. Ten micrograms of proteins from each sample of cells was electrophoresed by SDS-PAGE on a 4–12% gradient gel. Western blotting was performed using a LI-COR Odyssey CLx Infrared Imaging System (LI-COR Biosciences, Lincoln, NE, USA). The antibodies used were goat polyclonal anti-Rad53 (yC-19: sc6749, Santa Cruz Biotechnology, Santa Cruz, CA, USA) and rabbit polyclonal anti-HA (H6908, Sigma-Aldrich, Burlington, MA, USA). The secondary antibodies used were LI-COR IRDye 800CW goat polyclonal anti-rabbit IgG (H+L) 926-32211 and LI-COR IRDye 800CW donkey anti-goat IgG (H+L) 926-32214.

### 4.4. Analysis of the Topology of the Yeast 2-Micron (2μ) Plasmid

Yeast cells were grown in liquid SC medium to the late log phase. Nucleic acids were isolated from the cells using the glass bead method and fractionated on a 0.8% agarose gel in 0.5 × TPE (45 mM Tris, 45 mM phosphate, 1 mM EDTA, pH 8.0) supplemented with 12 μg/mL chloroquine. After Southern blotting, the membrane was subjected to hybridization with a radioactively labeled fragment of the 2μ plasmid as the probe and scanned on a phosphorimager to reveal the topoisomers of the 2μ plasmid. Under the electrophoresis conditions used in this work, more negatively supercoiled 2μ topoisomers migrated more slowly.

## 5. Conclusions

Hmo1 has diverse functions in genome structure and function. In this work, we presented genetic evidence implicating Hmo1 in the execution or regulation of multiple genome integrity mechanisms important for DDT, including homology-directed DNA repair, replication-coupled chromatin assembly, and the DNA damage checkpoint. Hmo1 may modulate the formation, stability, and/or removal of joint DNA structures such as SCJ or dHJ generated at stalled DNA replication forks directly by binding to these structures or indirectly by affecting chromatin assembly or structures that influence the formation or stability of joint DNA structures. In doing so, Hmo1 likely impacts multiple processes/steps of DDT (Figure 1), which should be taken into consideration when interpreting the effect of *hmo1Δ* on DDT. A key question regarding the molecular mechanism underlying Hmo1’s function in DDT is whether it depends on the known ability of Hmo1 to bend DNA, alter the nucleosome structure, compact chromatin, facilitate DNA supercoiling, or promote the DNA damage checkpoint. We obtained evidence that the contribution of the CTD of Hmo1 and by inference its DNA bending activity to DDT is dependent on the genetic background of the host. Of particular interest is our finding of an antagonistic relationship between Hmo1 and Htz1 in DDT that is correlated with their opposing impacts on DNA resection involved in DSB repair. Moreover, this relationship is dependent on the Hmo1 CTD. It will be interesting to examine whether Hmo1 and Htz1 also counteract each other to regulate DNA resection involved in DDT and how DNA bending promoted by the Hmo1 CTD facilitates Hmo1 to antagonize the function of Htz1.

## Data Availability

The data supporting the reported results are available to researchers upon request.

## Supplementary Materials

The following supporting information can be downloaded at https://www.mdpi.com/article/10.3390/ijms26073255/s1, References [47,62,82,83] are cited in the supplementary materials.
