# Supplementary material for: The Yeast HMGB Protein Hmo1 Is a Multifaceted Regulator of DNA Damage Tolerance
Source: Int J Mol Sci. 2025 Apr 1;26(7):3255. doi: 10.3390/ijms26073255 (PMC11989408; doi:10.3390/ijms26073255)
Supplement: Supplementary file 1 [file ijms-26-03255-s001.zip › ijms-3536576-supplementary.pdf]

**Supplementary Materials.**

**Table S1. Yeast strains used in this study.**

| # | Name | Genotype | Source/Reference |
|---|---|---|---|
| 1 | BY4741 | *MATa his3Δ1 leu2Δ0 met15Δ0 ura3Δ0* | Open Biosystems |
| 2 | BY-hmo1 | BY4741, *hmo1Δ::NatMX* | This work |
| 3 | BY-rad5 | BY4741, *rad5Δ::KanMX* | Open Biosystems |
| 4 | BY-rad5-h | BY4741, *rad5Δ::KanMX, hmo1Δ::NatMX* | This work |
| 5 | BY-mph1 | BY4741, *mph1Δ::KanMX* | Open Biosystems |
| 6 | BY-mph1-h | BY4741, *mph1Δ::KanMX, hmo1Δ::NatMX* | This work |
| 7 | BY-rad51 | BY4741, *rad51Δ::KanMX* | Open Biosystems |
| 8 | BY-rad51-h | BY4741, *rad51Δ::KanMX, hmo1Δ::NatMX* | This work |
| 9 | BY-rad52 | BY4741, *rad52Δ::KanMX* | Open Biosystems |
| 10 | BY-rad52-h | BY4741, *rad52Δ::KanMX, hmo1Δ::NatMX* | This work |
| 11 | BY-shu1 | BY4741, *shu1Δ::KanMX* | Open Biosystems |
| 12 | BY-shu1-h | BY4741, *shu1Δ::KanMX, hmo1Δ::NatMX* | This work |
| 13 | BY-rad54 | BY4741, *rad54Δ::KanMX* | Open Biosystems |
| 14 | BY-rad54-h | BY4741, *rad54Δ::KanMX, hmo1Δ::NatMX* | This work |
| 15 | BY-rdh54 | BY4741, *rdh54Δ::KanMX* | Open Biosystems |
| 16 | BY-rdh54-h | BY4741, *rdh54Δ::KanMX, hmo1Δ::NatMX* | This work |
| 17 | BY-sgs1 | BY4741, *sgs1Δ::KanMX* | Open Biosystems |
| 18 | BY-sgs1-h | BY4741, *sgs1Δ::KanMX, hmo1Δ::NatMX* | This work |
| 19 | BY-rmi1 | BY4741, *rmi1Δ::KanMX* | Open Biosystems |
| 20 | BY-rmi1-h | BY4741, *rmi1Δ::KanMX, hmo1Δ::NatMX* | This work |
| 21 | BY-top3 | BY4741, *top3Δ::KanMX* | This work |
| 22 | BY-top3-h | BY4741, *top3Δ::KanMX, hmo1Δ::NatMX* | This work |
| 23 | W1588-4C | *MATa ade2-1 can1-100 his3-11,15 leu2-3,112 trp1-1 ura3-1 RAD5+* | Ref. [82] |
| 24 | WY-hmo1 | W1588-4C, *hmo1Δ::NatMX* | This work |
| 25 | T382-P4 | W1588-4C, *smc6-P4-13-myc::H* | Ref. [82] |
| 26 | TY-hmo1 | W1588-4C, *smc6-P4-13-myc::H, hmo1Δ::NatMX* | This work |
| 27 | BY-mus81 | BY4741, *mus81Δ::KanMX* | This work |
| 28 | BY-mus81-h | BY4741, *mus81Δ::KanMX, hmo1Δ::NatMX* | This work |
| 29 | BY-mms4 | BY4741, *mms4Δ::KanMX* | This work |
| 30 | BY-mms4-h | BY4741, *mms4Δ::KanMX, hmo1Δ::NatMX* | This work |
| 31 | BY-slx4 | BY4741, *slx4Δ::KanMX* | This work |
| 32 | BY-slx4-h | BY4741, *slx4Δ::KanMX, hmo1Δ::NatMX* | This work |
| 33 | BY-rtt107 | BY4741, *rtt107Δ::KanMX* | Open Biosystems |
| 34 | BY-rtt107-h | BY4741, *rtt107Δ::KanMX, hmo1Δ::NatMX* | This work |
| 35 | BY-srs2 | BY4741, *srs2Δ::KanMX* | Open Biosystems |
| 36 | BY-srs2-h | BY4741, *srs2Δ::KanMX, hmo1Δ::NatMX* | This work |
| 37 | BY-pol32 | BY4741, *pol32Δ::KanMX* | Open Biosystems |
| 38 | BY-pol32-h | BY4741, *pol32Δ::KanMX, hmo1Δ::NatMX* | This work |
| 39 | PY39 | *MATα ura3-52 trp1Δ901 leu2-3,112 can1 pol30Δ1* [pBL230 (*POL30-TRP1*)] | Ref. [47] |
| 40 | PY39-h | PY39, *hmo1Δ::NatMX* | This work |
| 41 | PY39-79 | *MATα ura3-52 trp1Δ901 leu2-3,112 can1 pol30Δ1* [pBL230 (*pol30-79-TRP1*)] | Ref. [47] |
| 42 | PY39-79-h | PY39-79, *hmo1Δ::NatMX* | This work |
| 43 | BY-ctf4 | BY4741, *ctf4Δ::KanMX* | Open Biosystems |
| 44 | BY-ctf4-h | BY4741, *ctf4Δ::KanMX, hmo1Δ::NatMX* | This work |
| 45 | BY-dpb3 | BY4741, *dpb3Δ::KanMX* | Open Biosystems |

| | | | |
|---|---|---|---|
| 46 | BY-dpb3-h | BY4741, *dpb3Δ::KanMX, hmo1Δ::NatMX* | This work |
| 47 | BY-mrc1 | BY4741, *mrc1Δ::URA3* | This work |
| 48 | BY-mrc1-h | BY4741, *mrc1Δ::URA3, hmo1Δ::NatMX* | This work |
| 49 | BY-asf1 | BY4741, *asf1Δ::KanMX* | Open Biosystems |
| 50 | BY-asf1-h | BY4741, *asf1Δ::KanMX, hmo1Δ::NatMX* | This work |
| 51 | BY-rtt109 | BY4741, *rtt109Δ::KanMX* | Open Biosystems |
| 52 | BY-rtt109-h | BY4741, *rtt109Δ::KanMX, hmo1Δ::NatMX* | This work |
| 53 | YXB1543-1 | *MATa ura3 his3 ade2 can1 trp1 his5 LEU2-GAL10-FLP hhf1Δ::HIS3 hhf2Δ::LEU2 hht1Δ::LoxP hht2Δ::LoxP FRT-hml::URA3* [cir°] + pRS414-*HHT2-HHF2* | This work |
| 54 | YXB1543-1h | YXB1543-1, *hmo1Δ::NatMX* | This work |
| 55 | YXB1543-2 | *MATa ura3 his3 ade2 can1 trp1 his5 LEU2-GAL10-FLP hhf1Δ::HIS3 hhf2Δ::LEU2 hht1Δ::LoxP hht2Δ::LoxP FRT-hml::URA3* [cir°] + pRS414-*hht3-K56R-HHF2* | This work |
| 56 | YXB1543-2h | YXB1543-2, *hmo1Δ::NatMX* | This work |
| 57 | BY-rtt101 | BY4741, *rtt101Δ::KanMX* | Open Biosystems |
| 58 | BY-rtt101-h | BY4741, *rtt101Δ::KanMX, hmo1Δ::NatMX* | This work |
| 59 | BY-mms1 | BY4741, *mms1Δ::KanMX* | Open Biosystems |
| 60 | BY-mms1-h | BY4741, *mms1Δ::KanMX, hmo1Δ::NatMX* | This work |
| 61 | BY-mms22 | BY4741, *mms22Δ::KanMX* | Open Biosystems |
| 62 | BY-mms22-h | BY4741, *mms22Δ::KanMX, hmo1Δ::NatMX* | This work |
| 63 | BY-cac1 | BY4741, *cac1Δ::KanMX* | Open Biosystems |
| 64 | BY-cac1-h | BY4741, *cac1Δ::KanMX, hmo1Δ::NatMX* | This work |
| 65 | BY-cac2 | BY4741, *cac2Δ::KanMX* | Open Biosystems |
| 66 | BY-cac2-h | BY4741, *cac2Δ::KanMX, hmo1Δ::NatMX* | This work |
| 67 | BY-rtt106 | BY4741, *rtt106Δ::KanMX* | Open Biosystems |
| 68 | BY-rtt106-h | BY4741, *rtt106Δ::KanMX, hmo1Δ::NatMX* | This work |
| 69 | PY39-8 | *MATα ura3-52 trp1Δ901 leu2-3,112 can1 pol30Δ1* [pBL230 (*pol30-8-TRP1*)] | Ref. [47] |
| 70 | PY39-8-h | PY39-8, *hmo1Δ::NatMX* | This work |
| 71 | H3H4-1 | *MATa his3Δ200 leu2Δ0 lys2Δ0 trp1Δ63 ura3Δ0 met15Δ0 can1::MFA1pr-HIS3 hht1-hhf1::NatMX4 hht2-hhf2::[HHTS-HHFS]-URA3* | Open Biosystems |
| 72 | H3H4-1-h | H3H4-1, *hmo1Δ::NatMX* | This work |
| 73 | H3H4-2 | *MATa his3Δ200 leu2Δ0 lys2Δ0 trp1Δ63 ura3Δ0 met15Δ0 can1::MFA1pr-HIS3 hht1-hhf1::NatMX4 hht2-hhf2::[HHTS-hhf-L97A]-URA3* | Open Biosystems |
| 74 | H3H4-2-h | H3H4-2, *hmo1Δ::NatMX* | This work |
| 75 | BY-htz1 | BY4741, *htz1Δ::KanMX* | Open Biosystems |
| 76 | BY-htz1-h | BY4741, *htz1Δ::KanMX, hmo1Δ::NatMX* | This work |
| 77 | BY-swr1 | BY4741, *swr1Δ::KanMX* | Open Biosystems |
| 78 | BY-swr1-h | BY4741, *swr1Δ::KanMX, hmo1Δ::NatMX* | This work |
| 79 | BY-ino80 | BY4741, *ino80Δ::KanMX* | Open Biosystems |
| 80 | BY-ino80-h | BY4741, *ino80Δ::KanMX, hmo1Δ::NatMX* | This work |
| 81 | BY-AB | BY4741, *hmo1-AB-URA3* | This work |
| 82 | BY-rad5-AB | BY4741, *hmo1-AB-URA3, rad5Δ::KanMX* | This work |
| 83 | BY-rtt107-AB | BY4741, *hmo1-AB-URA3, rtt107Δ::KanMX* | This work |
| 84 | BY-slx4-AB | BY4741, *hmo1-AB-URA3, slx4Δ::KanMX* | This work |
| 85 | BY-srs2-AB | BY4741, *hmo1-AB-URA3, srs2Δ::KanMX* | This work |
| 86 | BY-cac2-AB | BY4741, *hmo1-AB-URA3, cac2Δ::KanMX* | This work |
| 87 | BY-htz1-AB | BY4741, *hmo1-AB-URA3, htz1Δ::KanMX* | This work |

| | | | |
|---|---|---|---|
| 88 | BY-swr1-AB | BY4741, *hmo1-AB-URA3, swr1Δ::KanMX* | This work |
| 89 | QY364 | *MATa hoΔ hml::ADE1 hmr::ADE1 ade1–100 leu2–3,112 trp1::hisG lys5 ura3–52 ade3::GAL::HO RAD9-HA-KanMX6* | Ref. [83] |
| 90 | QY364-h | QY364, *hmo1Δ::NatMX* | This work |
| 91 | QY375 | QY364, *hta1-S129*, hta2-S129** | Ref. [83] |
| 92 | QY375-h | QY375, *hmo1Δ::NatMX* | This work |
| 93 | PWT-1 | BY-hmo1 + pHWT[1] | This work |
| 94 | PWT-2 | BY-hmo1 + pRS416 | This work |
| 95 | PWT-3 | BY-hmo1 + pHAB[2] | This work |
| 96 | PRAD5-1 | BY-rad5-h + pHWT | This work |
| 97 | PRAD5-2 | BY-rad5-h + pRS416 | This work |
| 98 | PRAD5-3 | BY-rad5-h + pHAB | This work |
| 99 | PRTT107-1 | BY-rtt107-h + pHWT | This work |
| 100 | PRTT107-2 | BY-rtt107-h + pRS416 | This work |
| 101 | PRTT107-3 | BY-rtt107-h + pHAB | This work |
| 102 | PHTZ1-1 | BY-htz1-h + pHWT | This work |
| 103 | PHTZ1-2 | BY-htz1-h + pRS416 | This work |
| 104 | PHTZ1-3 | BY-htz1-h + pHAB | This work |

[1,2] The pHWT and pHAB plasmids were described in Ref. [62].
